# Co-Pigmentation Mechanism and Thermal Reaction Kinetics of Mulberry Anthocyanins with Different Phenolic Acids

**DOI:** 10.3390/foods11233806

**Published:** 2022-11-25

**Authors:** Xiangyue Chen, Qunyu Gao, Sentai Liao, Yuxiao Zou, Jiangang Yan, Qian Li

**Affiliations:** 1 Guangdong Academy of Agricultural Sciences, Sericultural & Agri-Food Research Institute/Key Laboratory of Functional Foods, Ministry of Agriculture and Rural Affairs/Guangdong Key Laboratory of Agricultural Products Processing, Guangzhou 510610, China; 2 Carbohydrate Laboratory, School of Food Science and Engineering, South China University of Technology, Guangzhou 510640, China; 3 Perfect (Guangzhou) Co., Ltd., Guangzhou 510006, China

**Keywords:** co-pigmentation, mulberry anthocyanin, phenolic acids, UPLC-Q-TOF-MS/MS, molecular docking

## Abstract

Applying the intermolecular co-pigmentation to improve the stability of mulberry anthocyanins is an important co-pigment method. Seven co-pigments, ferulic acid (FA), caffeic acid (CA), *p*-hydroxybenzoic acid (HBA), protocatechuic acid (PA), gallic acid (GA), vanillic acid (VA) and vanillin (VN) were selected to investigate mulberry anthocyanin co-pigmentation thermal reaction kinetics. The strongest co-pigment reactions were observed for FA at a molar ratio of 1:20, pH 3.5 and 20 °C, with the highest hyperchromic effects (52.94%), equilibrium constant (K) values (3.51) and negative values of Gibbs free energy (ΔG°) (−3.06 KJ/mol). Co-pigments that contained more free hydroxyl groups facilitated the co-pigmentation, and methyl contributed more to color enhancement, with respect to the hydrogen group. Ultra Performance Liquid Chromatography-Quadrupole-Time Of Flight-Mass/Mass Spectrometry (UPLC-Q-TOF-MS/MS) results indicated that FA and CA formed different anthocyanin derivatives with mulberry anthocyanin. The Fourier Transform Infrared Spectroscopy (FTIR) and molecular docking confirmed that hydrogen bonding, π–π stacking and hydrophobic interaction were formed between anthocyanins and three prevalent co-pigments (FA, CA and VA). CA and C3G could form four hydrogen bonds and two π–π stackings; this was the most stable system among three phenolic acid–C3G complexes. Due to the functional effect of phenolic acids, the addition of FA and CA not only enhanced the stability and color intensity of mulberry anthocyanins but also the functionality of the processing product.

## 1. Introduction

Mulberry is widely planted in China, Asia [1]; it is not only a high-value edible fruit but also widely used in food, medical, cosmetic and pharmaceutical industries [2,3]. Mulberry contains vitamins, flavanols, and phenolic acids and is rich in anthocyanins, one of the key bioactive ingredients extracted from mulberry [4]. Cyanidin-3-*O*-glucoside (C3G) and cyanidin-3-*O*-rutinoside (C3R) account for >98% of the total anthocyanin content [5,6,7]. Many biological activities have been confirmed for mulberry anthocyanins, such as antioxidant, antibacterial and anti-inflammatory [8,9]. Anthocyanins are often used as natural pigments in commercial food and beverage products because of their bright colors and functional activities [10]. However, as water-soluble pigments, the structure of anthocyanins is vulnerable to environmental factors, such as pH, temperature, light and oxygen. Other additive ingredients, such as enzymes, metal ions, oxides, sugar, etc., also affect the stability of anthocyanins during producing and processing process [11,12]. The instability of anthocyanins limits its industrial application in functional food, cosmetics and biomedicine fields [13].

To improve the stability of anthocyanin, the most common method includes intramolecular co-pigmentation and intermolecular co-pigmentation. Intramolecular co-pigmentation generally occurs between poly-acylated anthocyanins. The aromatic acyl groups of acylated anthocyanin molecules are folded by glycosylation, and the structure of anthocyanin is protected by the coplanar interaction and steric hindrance. There are several possible forms of intermolecular co-pigmentation; self-association, metal complexation, phenolic acid complex, protein complex and polysaccharides complex are the most common ways [14,15,16]. Phenolic acids were small in molecular weight, naturally green and functionally active; they are thereby widely used as co-pigments in the processing of beverages and ciders, which contain anthocyanins [10]. Previous studies have proved that many organic acids could cause significant hyperchromic effects and bathochromic shift; only two to three phenolic acids were selected for the comparison, including caffeic acid, *p*-coumaric acid, ferulic acid, etc. [17], while a few studies focused on the structure of organic acids, exploring the influence of different function groups and structures on co-pigmentation. In addition, the research on the mechanism and pathway of co-pigmentation between phenolic acids and anthocyanins does not go particularly deep. As common phenolic acids in many plants [18], ferulic acids (FA) are the most abundant hydroxycinnamic acid, with antioxidant [19] and anti-inflammation effects [20] and the ability to lower blood pressure [21], enhance vascular function [22] and even prevent the new coronary pneumonia (COVID-19) [23]. Gallic acid (GA) is one of the main active ingredients of Chinese traditional herbal medicine quintocin, which has powerful anti-allergic properties [24]. Caffeic acid (CA), protocatechuic acid (PA), P-hydroxybenzoic acid acid (HBA), vanillic acid (VA) and vanillin (VN) are natural secondary metabolites of plants [25], which have been reported to have anti-inflammatory, antibacterial, antiviral, and protective effects on cardiovascular diseases [26].

Based on differences in chemical structures, FA, CA, HBA, PA, GA, VA and VN, seven phenolic acids/aldehydes derived from natural plants, were selected to investigate co-pigmentation thermal reaction kinetics for mulberry anthocyanin. The co-pigmentation effect of hydroxyl and methyl on the organic acids benzene ring were compared in the present study. Furthermore, the UPLC-Q-TOF-MS/MS, FTIR and molecular docking were applied to analyze the product of co-pigmentation and explore the interaction force between anthocyanins and phenolic acids.

## 2. Material and Methods

### 2.1. Materials

Mulberry anthocyanins were bought from Tianjin Jianfeng Natural Products Research and Development Co., Ltd.(Tianjin, China) (250 mg/g). Ferulic acid, caffeic acid, *p*-hydroxybenzoic acid, protocatechuic acid, gallic acid, vanillic acid, vanillin phosphoric acid, anhydrous sodium acetate and sodium hydroxide were of analytical grade and purchased from Shanghai Ruiyong Biological Co., Ltd. (Shanghai, China).

### 2.2. Preparation of Co-pigmented Anthocyanin Solution

The co-pigmentation experiments referred to the method of Molaeafard, with some modifications [17]. The anthocyanin solution (2.5 × 10^−4^ M) was deployed in phosphate-buffered solution with the mixture of sodium acetate (0.02 M) and phosphoric acid (0.06 M). The pH of the buffer was adjusted to 3.5 using 1 M HCl or NaOH. FA, CA, HBA, PA, GA, VA and VN were used as co-pigments. Each of the co-pigments were dissolved in 12.5% ethanol phosphate-buffered solution to help the enhancement of the solubility in aqueous solution.

#### 2.2.1. The Effect of the Anthocyanin/Co-pigment Molar Ratio

The effect of the anthocyanin/co-pigment molar ratio was performed by mixing anthocyanin solution with seven co-pigments solution to obtain ratios of 1:0, 1:2.5, 1:5, 1:10, 1:20. The co-pigmentation was carried out at pH 3.5, 20 °C for 30 min in dark place. In addition, the stoichiometric ratio (*n*), equilibrium constant (*K*), and Gibbs free energy (Δ*G*°) of the co-pigmentation reactions were calculated using following equations [27,28,29]:

(1) $$\ln\left(\frac{A-A_{0}}{A_{0}}\right)=lnK+nln{\left(C_{P}\right)}_{0}$$

(2) ∆G=−RTlnK

where *A_0_* is the absorbance of natural mulberry anthocyanin (*λ*_516_), A is the absorbance of anthocyanin containing phenolic compounds (*λ*_516_), *K* is the equilibrium constant of the co-pigmentation reaction, *n* is the stoichiometric ratio between anthocyanin and co-pigment in the complex, *(C_p_)_0_* demonstrates the concentration of co-pigments, *R* is the global gas constant (8.3145 J/mol.K) and *T* is the temperature in Kelvin.

#### 2.2.2. The Effect of the pH on Co-pigmentation

The effect of the pH on co-pigmentation was performed under the condition of 1:20 anthocyanin/co-pigment molar ratio. The pH value of the samples varied in the range of 2.5–5.5, which was adjusted by addition of 1 M HCl or NaOH.

#### 2.2.3. The Effect of the Temperature on Co-pigmentation

The effect of the temperature on co-pigmentation was performed under the condition of 1:20 anthocyanin/co-pigment molar ratio, pH 3.5. Different temperatures were employed in a water bath for 30 min and the temperature range was from 20 to 80 °C, with intervals of 20 °C.

### 2.3. Thermal Degradation Kinetic Calculation

The mulberry anthocyanins solution and co-pigmented mulberry anthocyanins at molar ratio 1:20, pH 3.5, were heated in 70, 80, 90 °C constant temperature water bath for 250 min and the samples were selected every 50 min to determine A_λmax_; thus, the degradation rate constant k and half-life T_1/2_ were calculated according to the following equation:

(3) $$Ln\frac{A_{t}}{A_{0}}=-kt$$

(4) $$T_{1/2}=\frac{Ln2}{k}$$

where *At* is A_λmax_ of the sample solution after heating *t* min, *A*_0_ is A_λmax_ of the sample solution before heating, *t* is the heating time.

### 2.4. Ultra-Performance Liquid Chromatography–Tandem Mass Spectrometry

UPLC-Q-TOF-MS/MS was applied to explore the reaction product of the co-pigmentation. Cyanidin-3-*O*-glucoside (C3G) is the monomer with the highest proportion in mulberry anthocyanins. The co-pigmentation was performed by mixing C3G solution with seven co-pigments at 1:20 anthocyanin/co-pigment molar ratio, pH = 3.5, 20 °C. UPLC-Q-TOF-MS/MS analysis referred to the method of Khalifa, with some modifications [30] (Triple TOF 5600 high-resolution accurate-mass quadrupole time-of-flight mass spectrometer (AB Sciex) coupled to an ekspert ultra-LC 110-XL system (AB Sciex) was applied). UPLC CSH C18 column (2.1 mm× 100 mm; 1.7 μm; Waters, Milford, AS, USA) at 30 °C, with injection volume of 2 μL and flow rate of 0.3 mL/min. Mobile phase A was 0.1% formic acid aqueous solution, and mobile phase B was methanol solution. The gradient program was as follows: 0–10 min, 5–20% B, reserved for 5 min; 15–30 min, 20–25% B, reserved for 5 min; 35–40 min, 25–33% B; 40–42 min, 33–5% B, reserved for 5 min; 42–47 min, 5% B. The facilities equilibrate for 5 min every 5 samples. Mass spectrometry conditions refers to Huang’s studies [31].

### 2.5. FTIR and UV–Vis Spectroscopy

The FTIR was applied to investigate the interaction force between C3G and co-pigments and the product of co-pigmentation. The reactions were performed at 1:20 anthocyanin/co-pigment molar ratio, pH = 3.5, then the obtained solutions were freeze-dried to powder.

### 2.6. Molecular Docking

The structure of FA, CA and VA, three phenolic acids with the best hyperchromic effects, and C3G were obtained on the website Chem Spider (accessed on 8 August 2022). In this study, AutoDock Vina 1.1.2 software (Scripps Research institute, Olson lab, San Diego, CA, USA)was used for molecular docking work. The Py Mol plugin center of mass.py was used to define the center of the C3G molecular mass as the docking box, and the side length of the box was set to 22.5 angstroms. In addition, ADFR suite 1.0 (Scripps Research institute, Olson lab, San Diego, CA, USA) was used to convert all processed small molecules to PDBQT format required for docking with AutoDock Vina 1.1.2. (Scripps Research institute, Olson lab, San Diego, CA, USA) When docking, the exhaustiveness of the global search is set to 32, and the rest of the parameters remain at the default settings. The output highest scoring docked conformation was considered to be the binding conformation for subsequent molecular dynamics simulations.

### 2.7. Molecular Dynamics

All-atom molecular dynamics simulations were performed based on the C3G and C3G–phenolic acid complexes obtained by the above docking as the initial structures, and the simulations were performed using AMBER18 software (Scripps Research institute, Olson lab, San Diego, CA, USA) [32].

## 3. Results and Discussion

### 3.1. The Influence of Anthocyanins/Co-Pigmentation Molar Ratio on Co-Pigmentation

**Figure 1:**
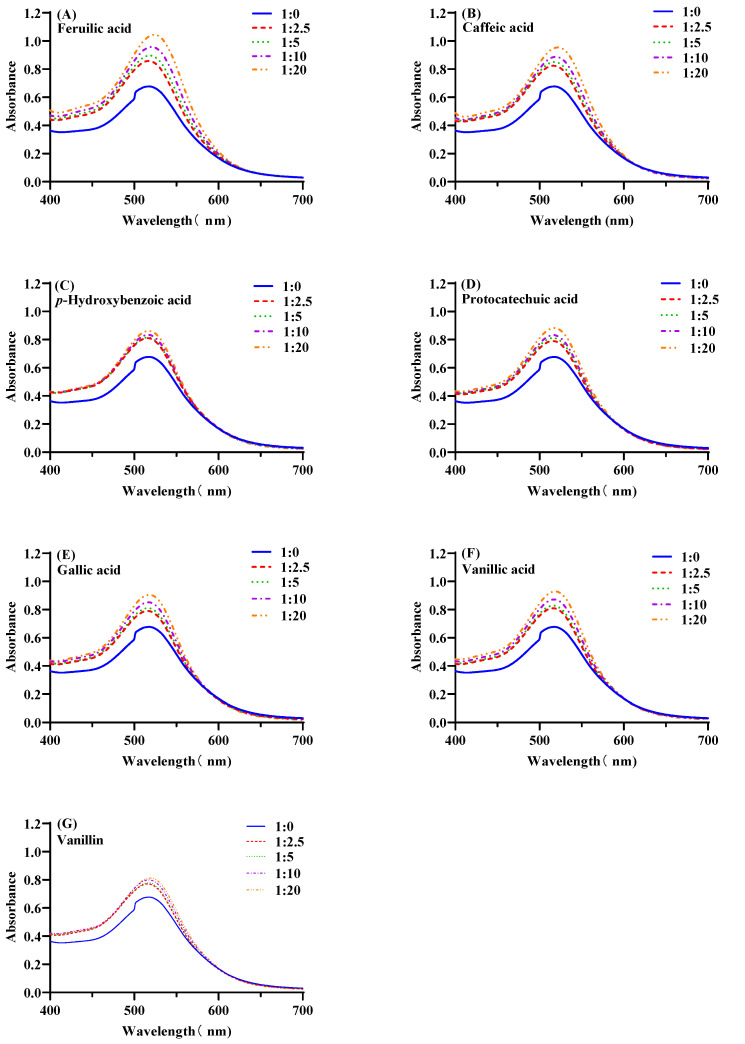
Visible absorption spectra of the mulberry anthocyanin co-pigmentation with different co-pigments and concentration at 20 °C and pH 3.5 in the range of 400–700 nm ((**A**): FA; (**B**): CA; (**C**): HBA; (**D**): PA; (**E**): GA; (**F**): VA; (**G**): VN).

**Figure 2:**
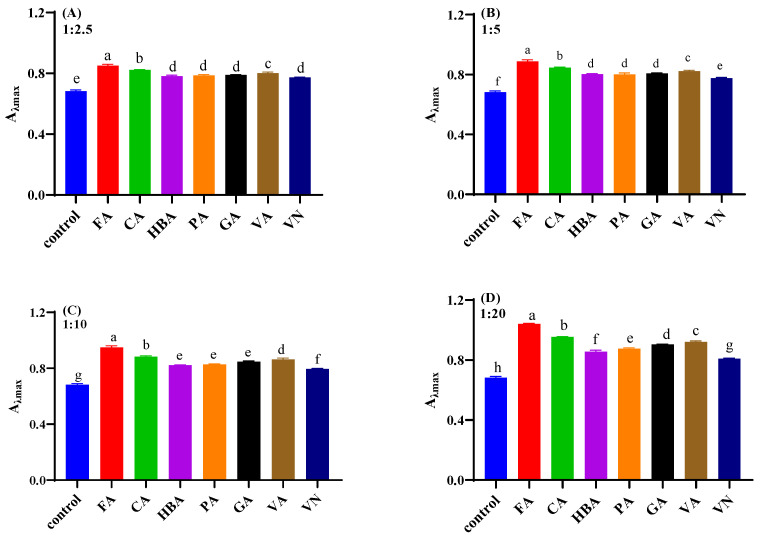
The A_λmax_ of the mulberry anthocyanin co-pigmentation at different molar ratio ((**A**) anthocyanin: co-pigments 1:2.5; (**B**) anthocyanin: co-pigments 1:5; (**C**) anthocyanin: co-pigments 1:10; (**D**) anthocyanin: co-pigments 1:20). Different letters indicate significant differences between samples (*p* < 0.05).

The visible absorption spectra of mulberry anthocyanin and co-pigmented mulberry anthocyanin by seven phenolic compounds at 20 °C and pH = 3.5, are shown in Figure 1. As the molar ratio increased from 1:2.5 to 1:20, the hyperchromic effects were increased correspondingly. Figure 2 exhibited the maximum absorbance of co-pigmented anthocyanins with different co-pigments. At the molar ratio 1:20, the adding of FA, CA, HBA, PA, GA, VA and VN had increased the maximum absorbance for 52.94%, 39.71%, 26.47%, 29.41%, 32.35%, 35.44% and 19.11%, respectively, compared with natural mulberry anthocyanin. In addition, the maximum wavelength of co-pigmented anthocyanin shifted from 516 nm to 517–520 nm. This result agreed with a previous study [33].

(5) $$AH^{+}⇋A^{+}+H^{+}$$

(6) $$AH^{+}+H_{2}O⇋\left(B+C_{E}\right)+H^{+}$$

(7) $$AH^{+}+nCP⇋AH{\left(CP\right)}_{n}$$

The above three equations existed in the anthocyanin and co-pigmentation system [34,35]. In the pH of 3–4, hemiketal B and the cis-chalcone *C_E_* are the main forms of anthocyanin structures. *AH^+^* is the flavylium ions and *CP* is the co-pigment. The addition of phenolic acids/aldehydes to mulberry anthocyanin would lead to a shift in the equilibrium towards the formation of complexes between mulberry flavylium ions and these co-pigments [36]. This mechanism would result in the enhancement of the solution color intensity and thereby enhance the solution absorbance [37].

FA, CA and VA (*p* < 0.05) had a better co-pigmentation effect compared with HBA, PA, GA and VN (*p* > 0.05) at a molar ratio of 1:2.5, while seven co-pigments all displayed their best co-pigmentation effect (*p* < 0.05) at a molar ratio of 1:20. Intermolecular co-pigmentation often occurred at high molar ratio [34,36,38]. The co-color effects of seven co-pigments were as follows: FA > CA > VA > GA > PA > HBA > VN. Benzene rings and carbon double bond were contained in both FA and CA, which could be the reason for the better co-pigmentation effect. Gallic acid had three hydroxyl groups attached to benzene rings that obtained the highest hyperchromic effects among GA, HBA and PA, which indicated that co-pigments contained more free hydroxyl groups facilitating co-pigmentation [17]. In addition, methyl contributed more to color enhancement with respect to the hydrogen group, based on the comparison of FA vs. CA and VA vs. GA [34,39]. Furthermore, all of the phenolic acids had better co-pigmentation effect than phenolic aldehydes, which were consistent with Xu’s study [40]. Overall, the co-pigment structure and variety significantly influenced the co-pigmentation effect on mulberry anthocyanin.

**Figure 3:**
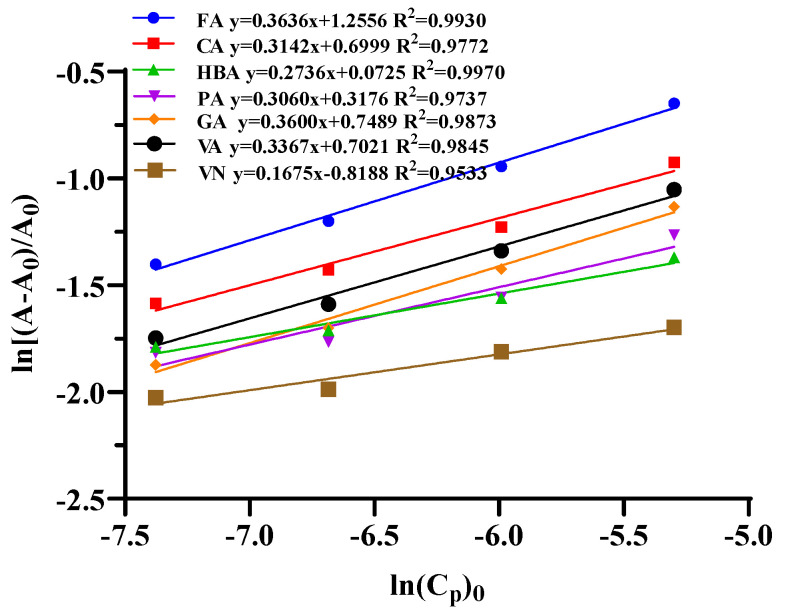
Plot of ln((A-A_0_)/A_0_) vs. ln(C*_P_*)_0_.

Plotting ln((A-A_0_)/A_0_) vs. ln(C*_P_*)_0_, a straight curve with slope n and intercept lnK, was obtained for each anthocyanin–co-pigment complex in Figure 3. The values of ΔG° were calculated with the substitution of ln K in Equations (1) and (2) and the ΔG° indicated the amounts of Gibbs free energy of the co-pigmentation process. The more negative the ΔG° value, the faster and more favorable the reaction was expected to occur. This order was consistent with the order of the co-pigmentation effect. The constant equilibrium values indicated the molecular bond strength between anthocyanin and co-pigment. Table 1 showed the n, K and ΔG° values for each co-pigmentation process. The ΔG° values were negative except vanillin, which indicated that all the co-pigmentation reactions in this investigation were spontaneous except vanillin. Ferulic acid, which had the most hyperchromic effect, showed the highest amount of equilibrium constant (3.51) and the highest negative value of Gibbs free energy (−3.66 KJ/mol), compared to other co-pigments. These results demonstrated that energy required for the co-pigmentation process for ferulic acid was the lowest, as compared to other groups. Vanillin obtained the biggest ΔG° and the lowest equilibrium constant, indicating the poorest hyperchromic effect.

The stoichiometric constant (*n*) was obtained by the plots of ln ((A–A_0_)/A_0_) vs. ln (C*_P_*)_0_, describing the association between the anthocyanin–co-pigment complexes. The *n* values of co-pigmentation complexes were about 0.17~0.36, which were lower than the previous studies [34]. The low stoichiometric constant (*n*) might be caused by the addition of ethanol in the co-pigmentation system, aiming to obtain a better solubility in water solution for phenolic acids/aldehydes. In addition, the results indicated that ethanol had a slight suppression effect to co-pigmentation.

### 3.2. The Influence of pH on Co-Pigmentation

**Figure 4:**
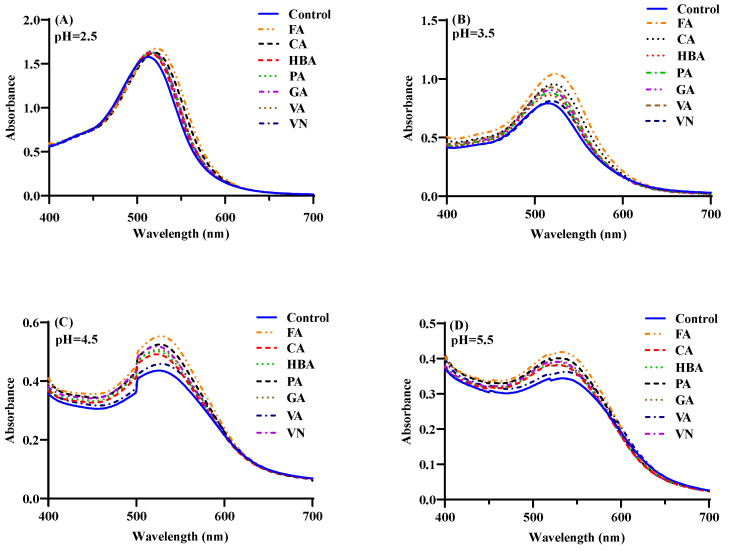
Visible absorption spectra of the mulberry anthocyanin co-pigmentation with co-pigments (1:20, 20 °C) at different pH value (**A**–**D**).

The complexation of mulberry anthocyanin with seven phenolic acids/aldehyde co-pigments was investigated under a pH that was from strong to moderate (pH 2.5–5.5). As shown in Figure 4, when the pH was 2.5, mulberry anthocyanin existed in the colored flavylium form [41], and its association with co-pigments only led to a slight bathochromic shift and increase in absorption at λ_516_ without significance. With the increase in pH, the visible spectrum of natural anthocyanin solution gradually declined, accompanied by changes in λ_max_, showing a clearer hypochromic effect compared with pH 2.5. Previous studies indicated that the co-pigmentation effect was more significant at pH around 3.6 than pH 2.5 [28,29], which was close to our result. The pH had a great influence on the structure of anthocyanins; hence, the noteworthy decrease in anthocyanins absorbance when the pH increased from 2.5 to 5.5 could be attributed to the formation of the colorless hemiketal form. It was believed that seven co-pigments relieved the hypochromic effects of pH, while the addition of co-pigments could make the equilibrium shift toward the colored form, thus enhancing the recovery ability against degradation [42]. At pH 3.5, the absorbance at λ_max_ increased by 2.40–29.67% under seven co-pigments, as compared with anthocyanins without additives.

In addition, although the seven co-pigments behaved in the same trend of increasing the absorbance, the magnitude of co-pigmentation effect was differently attributed to the different structure of co-pigments. It can be seen that at pH 3.5, FA, CA and VA were the three most effective phenolic acids to inhibit the degradation of anthocyanins, followed by GA, PA and HBA, while VN seemed to act as the relatively poor ones. This order was the same as the trend of the co-pigmentation effect.

### 3.3. The Influence of Temperature on Co-pigmentation

**Figure 5:**
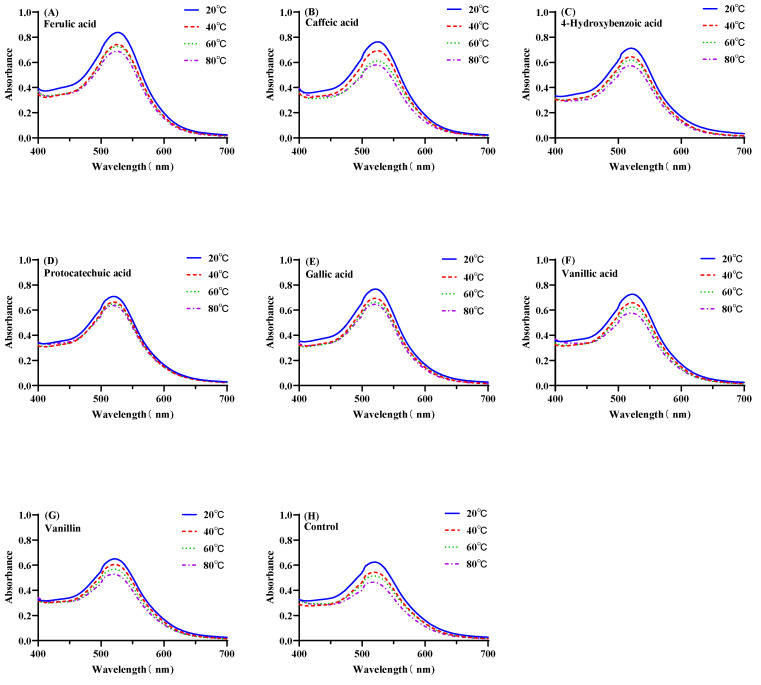
Visible absorption spectra of the mulberry anthocyanin co-pigmentation with co-pigments (1:20, pH = 3.5) at different temperatures ((**A**): FA; (**B**): CA; (**C**): HBA; (**D**): PA; (**E**): GA; (**F**): VA; (**G**): VN; (**H**): control).

Figure 5 shows the visible absorption spectra of mulberry anthocyanin with co-pigments at different temperatures in the range of 400–700 nm. The co-pigmentation reaction was investigated to illustrate the influence of temperature under the anthocyanin/co-pigments molar ratio of 1:20 and pH 3.5. Indeed, as the temperature increased from 20 to 80 °C, the reaction shifted toward the reactants, which destroyed the weak interactions of anthocyanin–co-pigment. Thus, the visible absorption spectra were progressively decreased in all of the anthocyanin–co-pigment complex samples.

### 3.4. The Co-Pigmentation Influence on the Thermal Degradation Kinetics of Mulberry Anthocyanins

**Figure 6:**
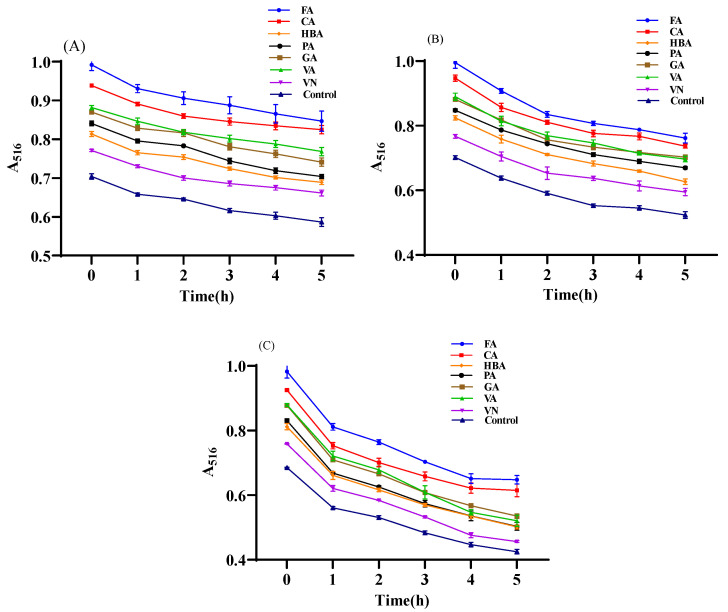
Thermal degradation of mulberry anthocyanins at different temperature ((**A**): 70 °C, (**B**): 80 °C, (**C**): 90 °C).

As shown in Figure 6, during the heating process, the maximum absorbance of the control group and the co-pigmented group decreased with the increase in heating time, and the higher the temperature, the faster the absorbance was decreased. This result was because high temperature can lead to the degradation of anthocyanins, thus reducing the content of mulberry anthocyanins. The thermal degradation kinetic parameters conformed to the first-order kinetic equation.

The data in Table 2 were calculated following the Formulas (3) and (4). The temperature has an important influence on the degradation of anthocyanins. As the temperature increased, the degradation constant (k) of the control and the co-pigmented groups increased, while the half-life (T_1/2_) decreased. This result was consistent with the research of Qian et al. [43]. In addition, at the same temperature, the degradation rate of co-pigment groups was lower and half-life was longer than that of the control group. It indicated that the thermal stability of mulberry anthocyanins improved after adding phenolic acids/aldehyde as co-pigments. Among seven co-pigments, FA, CA and VA, which had shown better hyperchromic effects, had better improvement on the stability of mulberry anthocyanins. The half-life of the CA group was the longest, which could be prolonged from 17.50 h to 31.28 h.

### 3.5. Component Analysis of Anthocyanin Derivatives by UPLC-Q-TOF-MS/MS

**Figure 7:**
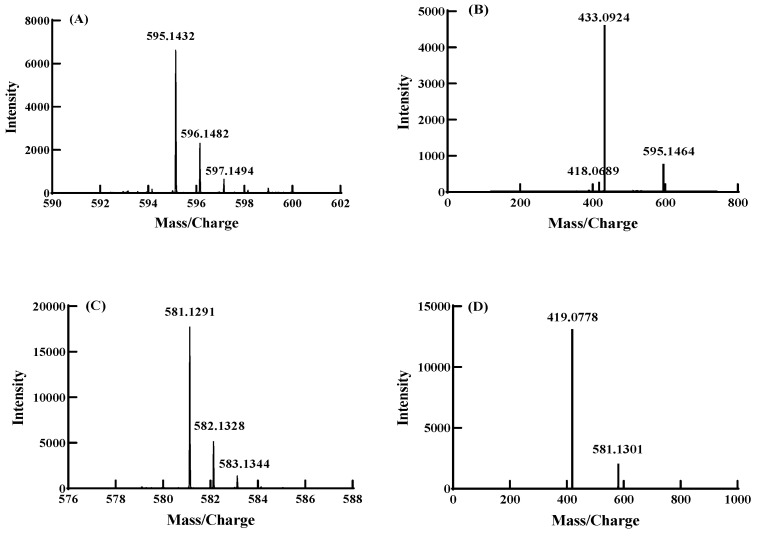
MS and MS/MS chromatograph of co-pigmentation product between ferulic acid/caffeic acid and C3G ((**A**): the MS chromatograph of FA–C3G complex; (**B**): the MS/MS chromatograph of FA–C3G complex; (**C**): the MS chromatograph of CA–C3G complex; (**D**): the MS/MS chromatograph of CA–C3G complex).

**Figure 8:**
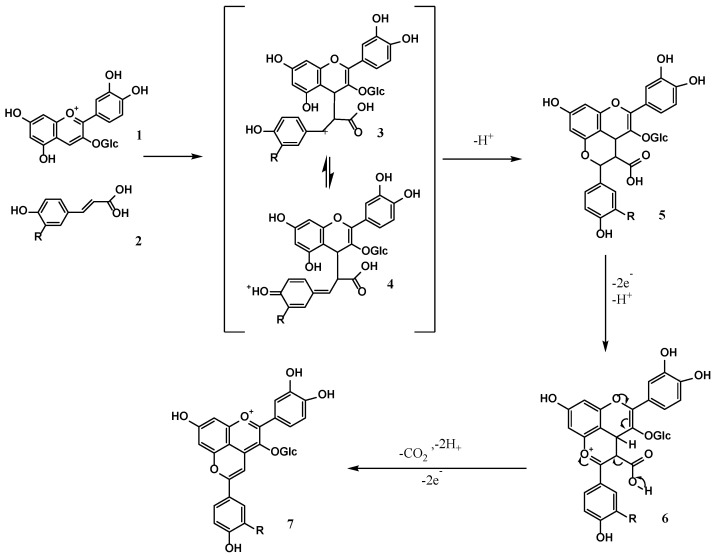
Formation pathway of anthocyanin derivatives between C3G and FA/CA.

Co-pigmentation between seven co-pigments and anthocyanins were investigated under molar ratio 1:20, pH 3.5 and 20 °C. UPLC-Q-TOF-MS/MS was applied to analyze the co-pigmentation product. The glucosides of anthocyanin and its derivatives were removed during the MS/MS spectrogram, which were used to identify the structures [44]. As shown in Figure 7, two major anthocyanin derivatives were found in co-pigmentation products of FA and CA, respectively, while no anthocyanin derivatives were detected in the co-pigmentation product solution of HBA, PA, GA, VA and VN. Figure 7A,B showed the first and second order mass spectra of the anthocyanin derivative formed with ferulic acid. It could be deduced that the molecular weight of anthocyanin derivatives formed with ferulic acid was 595.1432 from Figure 7A. The fragment ion at *m*/*z* 433.0924 in Figure 7B was likely obtained by removing glucose (162) from the molecular ion ([M]^+^ = *m*/*z* 595.1432). The molecular weight of the positive ion ([M]^+^ = *m*/*z* 595.1432) might be derived from the molecular weight of C3G (449) plus ferulic acid (194) and then minus 48 (44 + 4), following the pathway of forming anthocyanin–vinylphenol adducts in Figure 8 [40,45]. In a similar way, Figure 7C,D showed the first- and second-order mass spectra of the cyanidin-3-*O*-glucoside derivative formed with caffeic acid. The molecular weight of anthocyanin derivatives formed with caffeic acid was 581.1291 from Figure 7C and the difference between the molecular ion ([M]^+^ = *m*/*z* 581.1301) and fragment ion ([M]^+^ = *m*/*z* 419.0778) was exactly the molecular weight of the glucose (162). Similarly, the molecular weight of the positive ion was consistent with the molecular weight of C3G (449) plus caffeic acid (185) and then minus 48 (44 + 4), as shown in Figure 8.

The nuclear reactions were prone to start with the C-4 position of C3G, and FA and CA were typical nucleophiles. As a result, the initial bond formation between the C-4 position of C3G (1) and C-2 position of FA/CA due to the strong electrophilic nature of benzopyrylium unit and the nucleophilicity of acid α-carbon atom. Given the electron-deficient character of the resulting intermediate 3, it could be expected that electron-donating substituents on the aromatic ring of the cinnamic acid moiety facilitated this reaction due to the stabilization of intermediate carbenium ion 3. HBA, PA, GA, VA and VN had no structure of carbon double bonds conjugate with benzene rings; thus, the α-carbon atom did not exist, and intermediate 3 could not be formed. As a result, it could be speculated that there were two different ways of co-pigmentation between anthocyanins and phenolic acids/aldehydes. One was by forming anthocyanin–vinylphenol to increase the ability of anthocyanin against water, temperature and pH value. It has been confirmed that *p*-coumaric acid, ferulic acid, sinapic acid, and 4-dimethylaminocinnamic acid can be successfully reacted in this way [45]. The π–π interaction formed between the phenol rings and anthocyanins, hydrogen bonds between the anthocyanin and hydroxyl group attached to aromatic phenol rings, and charge transfer interactions were supposed to be another form of co-pigmentation between anthocyanins and phenolic acids/aldehydes. The path of forming anthocyanin–vinylphenol had a stronger co-pigmentation effect than forming hydrogen bonds; thus, in this study, FA and CA had a better co-pigmentation effect than other co-pigments.

### 3.6. FTIR Analysis

**Figure 9:**
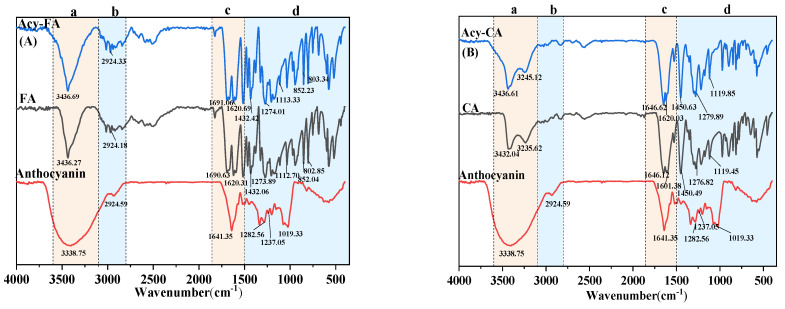

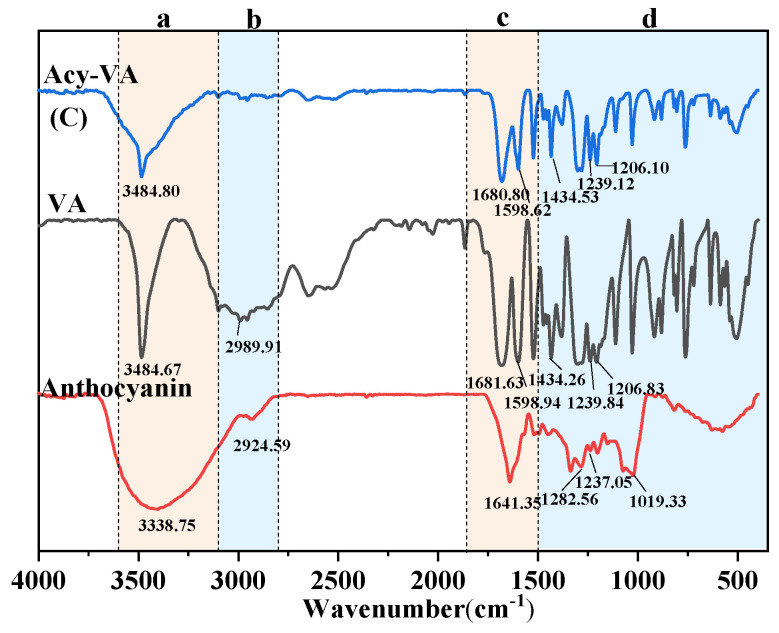
The Fourier transform infrared spectrum of anthocyanin derivatives co-pigmented between C3G and FA, CA, VA. (**A**–**C**) are the FTIR results of C3G-FA, C3G-CA, C3G-VA derivatives respectively.

FTIR spectroscopy was used to verify the two pathways of co-pigmentation between anthocyanins and phenolic acids. The FTIR spectroscopy of the three best co-pigmentation products (FA, CA and VA) are shown in Figure 9. A strong and broad absorption band at 3338 cm^−1^ was attributed to the stretching vibration of -OH, including the phenolic hydroxyl groups from anthocyanin, FA, CA, VA and alcoholic hydroxyl groups from the glycosyl of anthocyanins and water and hydrogen bonds. The peak at ~2831 cm^−1^ corresponded to the stretching vibrations of –CH_3_ and –CH_2_, and the peak at ~1641 cm^−1^ was caused by the vibrations of the benzene ring. The appearance of a peak at 1119 cm^−1^ correspond to the bending vibration C–O–C groups, and a band at 1262 cm^−1^ belong to stretching of pyran rings, typical for flavonoid compounds. The peak at 1119 cm^−1^ significantly strengthened after acylation with FA and CA, respectively, which were ascribed to the stretching vibrations of the C-O-C groups, as is shown in Figure 8 [46].

In the pathway of forming anthocyanin–vinylphenol in Figure 8, a new C-O-C group was formed, which could cause the enhancement of the peak at 1119 cm^−1^. It could be seen that the FTIR spectroscopy of anthocyanin reacted with VA and decreased significantly compared with pure VA, which was not present in FA and CA. This phenomenon might attribute to the hydrogen bonds formed between the anthocyanin and hydroxyl group attached to the aromatic phenol rings, which could cause the decreased absorption strength of the featured peak [47].

### 3.7. Molecular Dynamics Analysis

**Figure 10:**
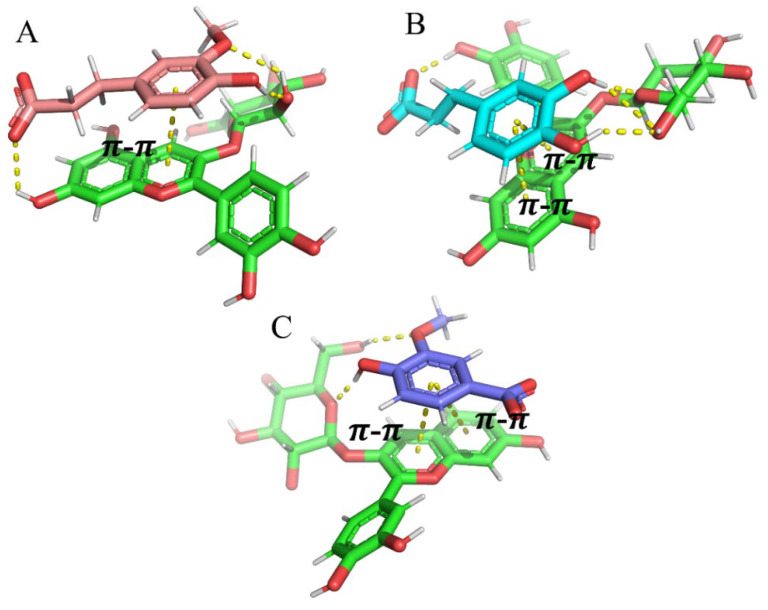
Molecular dynamic simulation obtained between C3G and three organic acids ((**A**) FA-C3G, (**B**) CA-C3G, (**C**) VA-C3G).

Molecular dynamics can simulate the interaction force between molecules and visualize the intermolecular force. To analyze the interaction force between C3G and three phenolic acids (FA, CA, VA), the present research docked the best conformation at 10 ns. As shown in Figure 10, the green molecular was C3G and hydrogen bonding and π–π stacking (pipi-stack) forces occurred between all the three phenolic acids and C3G. As shown in Figure 10A, three hydrogen bonds and a π–π stacking force occurred between C3G and FA. Hydrogen bond 1 occurred between the hydroxyl H atom at the C7 position of the C3G A ring and the FA carboxyl O atom, the length of which was 2.4 A. Hydrogen bond 2 occurred between the H atom from C3G glucoside and the O atom from FA methoxy, the length of which was 2.4 A. Hydrogen bond 3 occurred between the O atom from C3G glucoside and the H atom from FA phenolic hydroxyl group, the length of which was 2.0 A. As shown in Figure 10B, four hydrogen bonds and two π–π stacking forces occurred in the C3G–CA complex; hydrogen bond 1 occurred between the H atom from the phenolic hydroxyl group at the C3 position of C3G and the O atom of the CA carboxyl group, the length of which was 2.0 A. Hydrogen bond 2, 3 and 4 all occurred between the O atom from C3G glucoside and the H atom from the CA phenolic hydroxyl group, the lengths of which were 2.0, 2.6 and 2.2 A, respectively. As shown in Figure 10C, two hydrogen bonds and two π–π stacking forces occurred in the C3G–VA complex. Hydrogen bond 1 occurred between the H atom from C3G glucoside and the O atom from VA phenolic hydroxyl group, the length of which was 2.3 A. Hydrogen bond 2 occurred between the O atom from C3G glucoside and the H atom from the VA phenolic hydroxyl group, the length of which was 2.0 A.

Hydrogen bonding was one of the strongest interactions between molecules, which enhanced the stability of the C3G and phenolic acids complex. The results showed that FA, CA and VA improved the stability of C3G by forming hydrogen bonds and π–π stacking forces [48]; phenolic acids donated abundant electrons to anthocyanins, therefore, forming π–π stacking forces between C3G A ring, B ring and phenolic acids benzene ring [49]. It is worth mentioning that CA and C3G could form four hydrogen bonds; therefore, the C3G–CA complex was the most stable system among three phenolic acid–C3G complexes. CA had the best effect on reducing the thermal degradation constant of anthocyanins and prolonging its half-life, indicating that the combination of CA mulberry anthocyanins was more stable, which was consistent with the molecular docking results. FA had the best hyperchromic effects, indicating that hyperchromic effects were not only influenced by hydrogen bonds but also related to other intermolecular forces.

**Figure 11:**
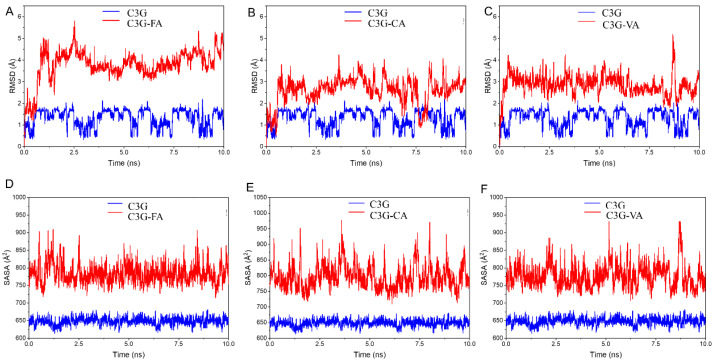
Changes in root mean square deviation (RMSD, (**A**–**C**)) and solvent accessible surface area (SASA, (**D**–**F**)) during molecular dynamic simulations.

The root mean square deviation (RMSD) of molecular dynamics simulation reflected the relative change between the initial conformation and the conformation at any time in the system. Severe fluctuations indicated violent motion; otherwise, the motion was smooth. As shown in Figure 11A–C, compared with pure C3G, the RMSD value of the C3G–phenolic acids complex had a narrower fluctuation range in the simulation process, which indicated that the system with the addition of phenolic acids was more favorable for the stability of C3G.

Solvent accessible surface area was a parameter of the molecular surface to solvent molecule interactions ratio, calculating the solvent accessible surface area could determine the changes in solvent accessibility of C3G residues, and thus predicted conformational changes during the binding degree. As shown in Figure 11D–F, The solvent accessible surface area of the complex of C3G and phenolic acid was larger than that of the pure C3G molecule, which meant that with the addition of FA, CA and VA, the complexes could interact with the solvent more easily, and thus the stability of the system would be further enhanced. The results indicated that the hydrophobic interaction of the solution might be one of the interactions between C3G and phenolic acids [50].

## 4. Conclusions

Co-pigmentation thermal reaction kinetics between seven phenolic acids/aldehydes and mulberry anthocyanins were compared in this study. Firstly, the most effective co-pigments, FA, CA and VA, were screened among seven phenolic compounds. Co-pigmentations were significantly influenced by the concentration of co-pigments, pH value, temperature and the chemical structure of co-pigments. The strongest co-pigment reactions were observed for FA at the molar ratio 1:20, pH 3.5 and 20 °C, with the highest hyperchromic effects (52.94%), equilibrium constant (K) values (3.51) and negative values of Gibbs free energy (ΔG°) (−3.06 KJ/mol). For chemical structure, more hydroxide groups were significantly benefited in forming hydrogen bonds with anthocyanins, and methyl groups led to better a co-pigmentation effect than hydroxide group, relatively speaking. Then, the FTIR and AutoDock data confirmed that hydrogen bonding, π–π stacking and hydrophobic interaction were formed between anthocyanins and three phenolic acids (FA, CA and VA). Four hydrogen bonds and two π–π stackings were formed between C3G and CA. In addition, due to the special conjugated structure of the benzene ring and double bond, FA and CA could form two different anthocyanin–vinylphenol, which improved the stability of the color intensity of anthocyanin by forming more stable molecules. This might the reason why FA and CA had the best co-pigmentation effect and stability. Overall, this study provided a green choice for mulberry anthocyanins color enhancement in the application of mulberry wine and other liquid beverages. Most importantly, our results provided a theoretical basis for the utilization of anthocyanins in food coloring.

## Figures and Tables

**Table 1:** Thermodynamic properties for the co-pigmentation reactions of mulberry anthocyanin with different co-pigments.

Co-Pigments	Equation	*n*	K	ΔG° (KJ/mol)
FA	y = 0.3636x + 1.2556 R^2^ = 0.9930	0.36	3.51	−3.06
CA	y = 0.3142x + 0.6999 R^2^ = 0.9772	0.31	2.01	−1.71
HBA	y = 0.2736x + 0.0725 R^2^ = 0.9970	0.27	1.08	−0.18
PA	y = 0.3060x + 0.3176 R^2^ = 0.9737	0.31	1.37	−0.77
GA	y = 0.3600x + 0.7489 R^2^ = 0.9873	0.36	2.11	−1.83
VA	y = 0.3367x + 0.7021 R^2^ = 0.9845	0.34	2.02	−1.71
VN	y = 0.1675x − 0.8188 R^2^ = 0.9533	0.17	0.44	2.00

**Table 2:** The thermal gradation kinetic parameter for of mulberry anthocyanins at different temperatures.

Co-pigments	T (℃)	k × 10^3^ (min^−1^)	T_1/2_ (h)
Control	70 °C	0.66 (0.9749)	17.5
	80 °C	0.95 (0.9389)	12.18
	90 °C	1.74 (0.9847)	6.66
FA	70 °C	0.47 (0.9986)	24.62
	80 °C	0.82 (0.9330)	14.13
	90 °C	1.22 (0.9527)	9.46
CA	70 °C	0.37 (0.9408)	31.28
	80 °C	0.71 (0.9598)	16.21
	90 °C	1.05 (0.9547)	10.98
HBA	70 °C	0.56 (0.9801)	20.48
	80 °C	0.91 (0.9884)	12.63
	90 °C	1.38 (0.9981)	8.35
PA	70 °C	0.60 (0.9829)	19.27
	80 °C	0.80 (0.9793)	14.5
	90 °C	1.44 (0.9971)	8.00
GA	70 °C	0.58 (0.9829)	19.77
	80 °C	0.71 (0.9072)	16.19
	90 °C	1.45 (0.9950)	7.98
VA	70 °C	0.46 (0.9847)	24.98
	80 °C	0.77 (0.9824)	14.99
	90 °C	1.45 (0.9902)	7.95
VN	70 °C	0.47 (0.9596)	24.55
	80 °C	0.80 (0.9564)	14.42
	90 °C	1.64 (0.9838)	7.04

## Data Availability

The data presented in this study are available on request from the corresponding author.
